# A risk assessment study of the occurrence and distribution of aflatoxigenic *Aspergillus flavus* and aflatoxin B1 in dairy cattle feeds in a central northern state, Nigeria

**DOI:** 10.1016/j.toxrep.2018.08.011

**Published:** 2018-08-16

**Authors:** G.K. Omeiza, J. Kabir, J.K.P. Kwaga, C.N. Kwanashie, M. Mwanza, L. Ngoma

**Affiliations:** a Department of Veterinary Public Health and Preventive Medicine, Faculty of Veterinary Medicine, University of Abuja, Nigeria; b Department of Veterinary Public Health and Preventive Medicine, Faculty of Veterinary Medicine, Ahmadu Bello University, Zaria, Nigeria; c Department of Veterinary Microbiology, Faculty of Veterinary Medicine, Ahmadu Bello University, Zaria, Nigeria; d Department of Animal Health, Faculty of Natural and Agricultural Sciences, Mafikeng Campus, North West University, Private Bag X2046, Mmabatho 2735, South Africa

**Keywords:** *Aspergillus flavus*, Aflatoxin B1, Occurrence, Dairy feeds, Risk, Nigeria

## Abstract

Nigeria, being a tropical nation, characterized by favorable climatic conditions, may display high chances of feed contaminations due to aflatoxigenic *Aspergillus flavus* with the consequences of health risks associated with the consumption of dairy products. A cross-sectional study was conducted to examine the risks of occurrence and distribution of aflatoxigenic *Aspergillus flavus* (*A. flavus*) and aflatoxin B1 (AFB1) contamination levels based on the European Union (EU) and United States Food and Drug Administration (USFDA) set limits. Feeds (n = 144) were collected from selected conventional and traditional dairy herds; prepared and analyzed using immuno-affinity column (IAC) and high performance liquid chromatography (HPLC) respectively. Forty eight (55.8%) isolates were identified as *A. flavus* of the isolated Aspergilli (n = 86). Of this proportion, 12 (25.0%) were aflatoxigenic strains. An outrageous number of the tested feeds (86.8%, n = 144) were positive for AFB1 contamination. Detectable AFB1 concentrations ranged between (0.5 and 24.8) μgKg^−1^ and were distributed variously according to many factors of distribution. Eighty three (66.4%, n = 125) of the AFB1 contaminated feed samples showed AFB1 concentrations between 5 and ≥20 μgKg^-1^. All-inclusive policies are key to reducing the health risks posed to the consumers of dairy products.

## Graphical abstract

**Figure:**
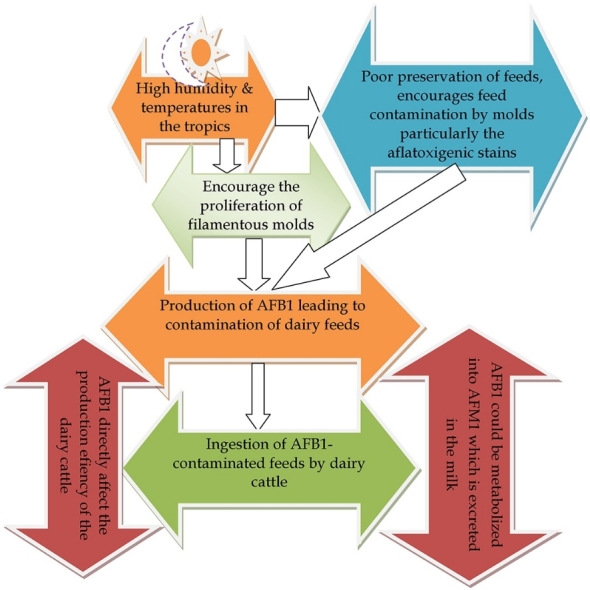

## 1 Introduction

Feeds and feeding are significant component of a successful animal rearing. Such importance is not unconnected to the roles feeds play in providing vital nutrients and energy. The latter attribute of feeds as indicated above is particularly significant in the area of animal production wherein feeds support the basic production needs and processes. One clear illustration of the dependence of animal production on rational feeding system is the dairy farming system where high energy demand is a requirement for optimal milk output.

Milk production is an energy-dependent processes. In Nigeria dairy production is gaining much attention because of the high demands placed on the dairy products by its timid uncontrolled population growth. Many dairy farms subsist on open grazing coupled with feed supplementation [21]. Most of the feeds used in dairy industry are principally compounded to meet the high energy requirement of the dairy cattle in terms of the quality of the feed dry matter and crude proteins meant to improve milk yield in both quality and volume.

Other considerations towards achieving optimal dairy outputs is breed selection amidst other factors such as proper feed management [3]. These aspects of the dairy sector have been well programmed in many parts of the world to achieve quality dairy products in terms of the quantity and safety. One of the major goals of the dairy industry is the high milk yield. This is achievable through a well organized management practices of the dairy industry. A good indicator of such an organized and efficient dairy performance is the yearly calving interval is an indication of high resulting from effective feed utilization [25]. Fortunately, the Nigerian Zebu breed of cattle has been selectively proven to possess the genetic capabilities and requirements for optimal dairy production if properly managed [22]. Good management of the dairy cattle depends solidly on good feeding plans amongst other vital considerations, and if this aspect of the management lacks proper management, it portends health risks and perhaps production wastage [3]. Such situation could be worst when dealing with feeds of concentrate origin known to contain high nutrient composition [41,38,17].

Molds particularly the *Aspergillus* section *Flavi,* are known contaminants of feeds and other food raw materials [52]. These organisms survive on the dry matter contents of the feeds particularly of the grains and other rich concentrates [38]. The dry matter contents of feeds are significantly reduced by mold infestations; implying a state of altered energy requirements of the feed with the consequences of poor crop yield [52]. Dairy animals feeding on feeds of reduced energy contents may suffer production loss [52,6], therefore requiring extra feed supplementation and cost [32]. It thus implies that formulation of meals based on estimated values without prior knowledge of the likely changes in the nutritional contents may lead to malnutrition and poor production [56].

Mold contamination of dairy feeds may also border around certain health and production risks, particularly in situations where the contaminating agents are capable of producing mycotoxins on dairy cattle feeds including concentrates [17,30]. Aflatoxin B1 is one of the most dreaded hepatotoxigenic mycotoxins. AFB1 is produced principally by the *Aspergillus flavus* and *A. parasiticus.* The presence of AFB1 in feeds of dairy cattle leads to the emergence of AFM1 in milk and dairy products [2,17]. The growth and proliferation of these agents are usually favored by certain tropical climatic conditions such as high temperature and humidity. In cattle, chronic ingestion of aflatoxins causes various adverse effects such as increased susceptibility to disease, loss of reproductive performance, and in case of dairy cattle, a decrease in yield and quality of milk production [50]. Aflatoxins, particularly AFB1, have been described in both acute and chronic forms [56].

Higher proportion of Nigerians are living below the poverty line with the consequences of malnutrition.

Due to the fast growing of the Nigerian population, the nutritional requirements have been increased and could shortly be achieved through high dairy yields. Nigerian cattle have been reported to have the right genetic potentials to produce more than enough milk for consumption and even for exportation. One limiting factor challenging the setting is the conducive climate which encourages the growth and proliferation of mold and their toxins [42]. It is against this background that a study was advanced to assess the risks of occurrence and distribution of aflatoxigenic *Aspergillus flavus* and aflatoxin B1 in dairy cattle feeds in a popularly located central Northern State of Nigeria.

## 2 Materials and methods

### 2.1 Selection of dairy farms for the study

The study was conducted in ten different dairy outfits sited in different locations of Kaduna State, comprising of 3 commercial, 3 institutional dairy farms and 4 traditional Fulani dairy herds belonging to the Fulani dairy Cooperative groups. Sampling began with the selection of dairy farms and herds actively engaged in dairy activities. Recruitment of farms and dairy herds for the study was strictly based on the current dairy records of milk production. At the time of the study many farms exist with no records of active production due to the experiences of cattle rustling at a time.

### 2.2 Feed sampling

The selected commercial, institutional and sedentary traditional Fulani dairy herds represent the sampling site. Feed samples were collected as fresh and preserved (stored) feeds (where applicable). Polytene bags and metal probes were purchased and sterilized for collection of the samples from feeding troughs and stores respectively. In the case of preserved bulk samples, systematic random sampling technique was adopted. An imaginary diagonal line was drawn across the stored bags of feeds. Bags were selected along the line with intervals of 3 bags in between them. The selected bags were probed each at different points to pool an estimated representative sample of averagely 20 g per farm/herd. In the case of fresh feeds, two feeding troughs were randomly selected among others in the milking parlour. Collected feed samples were pooled to make 20 g representative feed sample per farm/herd. Sampling continued weekly until a total pooled feed samples reached 144. For ethical reason, names of dairy farms and dairy herds used in this study were coded as Farm A (NP), Farm B (DC), Farm C (YS), Farm D (CG), Farm E (JM), Farm F (GG). Other cattle herds comprising of Fulani dairy herds (FH) were coded as EM, JN, AL and JE.

### 2.3 Phenotypic isolation and identification of the aflatoxigenic *Aspergillus flavus*

#### 2.3.1 Microbiological isolation

Sampled feeds were first analyzed for moisture contents using oven-drying method. The feed samples were subsequently investigated for the presence of *A. flavus* using a modified isolation method previously reported [16,20]. A total of 20 g of feed sample was collected, ground and homogenized from each farm out of which 1 g was prepared as a single-fold dilution in a test tubes using 9 ml sterile water. A sterile syringe was used to aspirate 1 ml of the feed suspension and dispensed on to a sterile SDA medium for culturing. At each time, a sterile spreader was employed to gently and evenly spread the dispensed feed suspension. The inoculated Petri dishes containing the samples were incubated at room temperatures between 25 and 30 °C in a relatively dark place for an average of 3 days. Suspicious colonies of *A. flavus* were identified by their greenish-yellow appearance and powdery texture with the reverse side pale to yellow [40]. Suspected colonies of *Aspergillus* spp were counted and presented as Log10 CFU/gram of feed according to an earlier described method [20]. Pure cultures of the suspects were obtained following repeated isolation and maintained as stock cultures using a water culture technique as previously described [5].

#### 2.3.2 Phenotypic identification on culture media

Primary macroscopic morphological studies were carried out on Sabouraud dextrose agar (SDA) while Czapek Dox Agar and Rose Bengal agar were used as differential media and Identification of *A. flavus* followed the method previously reported [12,48]. A locally prepared neutral red desiccated coconut agar (NRDCA) [43] was used to isolate and identify the aflatoxigenic isolates of the *A. flavus* under a long wavelength UV. The fluorescence characteristics of the of the isolates were observed and categorized into a very strong fluorescent, strong fluorescent, weak fluorescent and non-fluorescent samples indicative of their potentials of aflatoxin production.

#### 2.3.3 Microscopic identification

The microscopic features of the isolates were studied using lactophenol cotton blue staining technique [12,51]. A drop of the stain was placed on a clean slide. A small part of the fungal cultures was removed and placed in the drop of the dye using a mounting needle. The mycelium was then spread by the same needle. A cover slip was then gently placed on the spread mixture with gentle digitalpressure to eliminate air bubbles. The slide was then mounted and observed under X40 objective lens. Identification of *A. flavus* was based on the presence of septate hyphae, rough and colourless conidiophoreswhich end in vesicle having the entire surface covered with either uni- or biseriate sterigmata. Directly at the base of the vesicle, the conidiophores contain a dark spot.

#### 2.3.4 UV-based fluorescence identification of the aflatoxigenic strains

Identification of aflatoxigenic strains of the isolated *A. flavus* adopt a technique reported by [43]. All suspected colonies of *A. flavus* were sub-cultured on a locally prepared Neutral Red Desiccated Coconut Agar (NRDCA). The positively identified growths were viewed under a long wavelength (365 nm). Fluorescent characteristics were studied and reported as positive for those colonies that showed fluorescence and negative for the non-reactants.

### 2.4 Polymerase chain reaction methods used to identify the strains of *Aspergillus flavus*

#### 2.4.1 Fungal DNA extraction

The genomic DNA of the *A. flavus* was isolated using Fungal/Bacterial DNA extraction kit (Zymo Research Corporation, Southern California, USA) according to the manufacturer’s instructions. The fungal isolates were grown on PDA plates and 5-day old cultures were utilized for the extraction of the DNA. About 200 mg of the mycelia was harvested from the agar surface by the use of a sterile wire loopThe harvest was suspended in 750 μl of lysis solution contained in a 1.5 ml ZR Bashing Bead^™^ lysis tube, which was placed in disruptor genie bead beater fitted with a 2 ml tube holder assembly (Scientific industries Inc., USA) and processed at maximum speed for 5 min. This was followed by centrifugation of the lysed samples at 10,000 × *g* for 1 min. The supernatant was transferred to a Zymo-Spin^™^ IV spin filter in a 1.5 ml eppendorf tube and again centrifuged at 7000 × *g* for 1 min. The content was then filtered into a collection tube and 1200 μl of fungal/bacterial DNA binding buffer added and vortexed. The extracted mixture (800 μl) was transferred to a Zymo spin^™^ IIC column in the collection tube and again centrifuged at 10,000 *g* for 1 min and the supernatant discarded. An aliquot (200 μl) of DNA pre-wash buffer was then added to Zymo spin^™^ IIC column in a new collection tube and centrifuged at 1000 *g* for 1 min. The filtrate was discarded while retaining the column, which was placed into a new tube. A 500 μl aliquot of the DNA wash buffer was added to Zymo spin^™^ IIC column and again centrifuged at 10,000 × *g* for 1 min. Finally, the Zymo spin^™^ IIC column was transferred into a sterile 1.5 ml eppendorf tube and 100 μl DNA elution buffer was added directly to the column matrix, and was centrifuged at 10,000 × *g* for 30 s to elute the DNA. The eluted DNAs were purified and concentrations determined using micro volume spectrophotometer. The purified DNA was then stored at −80 °C for further analysis.

#### 2.4.2 Identification of *Aspergillus flavus*

The intergenic spacer region (IGS) of the fungal DNA homologous to the genus *Aspergillus* was amplified using a primer set : *Asp*-F, 5′-CGGC CCTTAAATAGCCCGGTC-3′; *Asp-*R, 5′-ACCCCCCTGAGCCAGTCCG-3′ encoding an amplicon size of 500 bp [24]. The IGS is located between V7 and V9 regions of the *18S rRNA* [54,24,47]. A specific primer set (*fla-*F., 5′ -GT A GGG TTC CT A GCG AGCC-3′; *fla*-R., 5′-GGA AAA AGA TTG ATT TGCG-3′) encoding an amplicon size of 500bp [1] was used to amplify certain flanking gene fragment *(fla)* specific to *Aspergillus flavus* specie located in the highly variable portion of the internal transcribed spacer regions, *ITS* [15]*.*

#### 2.4.3 Molecular differentiation between *A. flavus* and *A. parasiticus*

##### 2.4.3.1 Restriction site analysis of PCR products of IGS (PCR-RFLP)

The IGS which encloses the aflatoxin biosynthesis genes (*aflJ-aflR*) was the target for amplification in order to differentiate *A. flavus* from a morphologically and genetically related *A. parasiticus*. A primer pair IGS-F/IGS-R was used to amplify the available regions from those isolates [55] that correspond to a PCR product of 674 bp. The sequences of the primers used are as follows: IGSF-5′AAGGAATTCAGGAATTCTCAATTG3′;IGSR-5′GTCCACCGGCAAATCGCCGTGCG-3′. The PCR products were subjected to endonuclease restriction enzyme digestion using *Bg III* (Zymo Research Corporation, Southern California, USA). The reactions were performed in a total volume of 40 μl containing 15 units of enzyme, 4 μl of buffer, 15 μl of PCR product, and Ultrapure water up to 40 μl. The reaction mixture was incubated at 37 °C for 3 h. Then the resulting fragments were separated by electrophoresis on a 2% w/v agarose gel for 1 h 45 min at 100 V.

##### 2.4.3.2 PCR reactions

Individual PCR reactions contained 4 μl of DNA (12–116 ng /μl) template which was mixed with 25 μl master mix (Taq DNA polymerase (Fermentas Life Science, Lithuania), dNTPs, MgCl_2_ and reaction buffers at optimal concentrations for efficient amplification of DNA templates by PCR), 1 μl of the primer i.e. Reverse (0.5 μl), Forward (0.5 μl) and 20 μl of nuclease free water to make up a reaction volume of 50 μl A negative control containing all the reagents except the DNA was also prepared. The PCR was performed in eppendorf tubes placed in a C1000 Touch^™^thermocycler (Bio-Rad, USA). The conditions for PCR were as follows: initial denaturation of DNA at 95 °C for 3 min and then 35 cycles of denaturation at 94 °C for 1 min, primer reannealing at 58 °C for 45 s and extension at 72 °C for 1.5 min. The PCR was finally extended for 10 min at 72 °C and held at 4 °C until samples were retrieved.

##### 2.4.3.3 In vitro detection of genes that encode aflatoxin production

Three structural and one regulatory genes were used to identify aflatoxin-producing potentials amongst the isolated *Aspergillus flavus.* These include: norsolorinic reductase *(nor),* o-methyl transferase *(omt),* vesicolorin dehydrogenase *(ver)* and aflatoxin regulated gene *(aflR).* The Primers used have been previously described [46, 13, 1, 47, 37. The sequences of the primers are: *omt-*Fw, 5′-GTG GAC GGA CCT AGT CCG ACA TCAC-3′; *omt-*Rev, 5′-GTC GGC GCC ACG CAC TGG GTT GGGG-3′; *nor*-F, 5′-ACCGCT ACGCCGGCACTCTCGGCAC-3′; *nor*-R, 5′-GTTGGCCGCCAGCTTCGACACTCCG-3′;*ver-*F, 5′-GCCGCAGGCCGCGGAGAAAGTGGT-3′; *ver-*R, 5′ -GGGGAT ATACTCCCGCGACACAGCC-3′; *aflR-*F, 5′-TATCTCCCCCCGGGCATCTCCCGG-3′; *aflR-*R, 5′−CCGTCAGACAGCCACTGGACACGG-3′ [46,13,47,37]

##### 2.4.3.4 Gel electrophoresis of PCR products

Agarose gel DNA electrophoresis was performed according to the method previously described [35]. One time Tris/Acetate/ EDTA (1x TAE) buffer was prepared by adding 4900 ml of distilled water to 100 ml of 50x TAE (375 ml of Tris-Cl, 28.55 ml of acetic acid, 50 ml of EDTA and 46.45 distilled water) and filled in the electrophoresis tank. Two grams of agarose (Fermentas Life Science, Lithuania) was prepared in 98 ml of 1x TAE buffer to give a 2% solution which was melted in a microwave. The solution was allowed to cool to 60 °C prior to the addition of 3 μl of 2% ethidium bromide (Sigma-Aldrich, ST Louis, MO, USA) and thoroughly mixed. The gel was poured into the casting chamber (Bio-Rad laboratories, California, USA) and the combs of desired sizes were affixed in such a way that no bubbles were trapped under the teeth. After the gel was set, the combs were nbcxgently removed and the gel was placed in the electrophoresis tank. The PCR product (8 μl) mixed with 6 μl of loading dye was slowly loaded into each of the wells in the gel with sterile micro pipette. Care was taken not to cross-contaminate the wells. A 6 μl of molecular marker also referred to as Gene Ruler (1 kilo base (kb) DNA ladder (Fermentas Life Science, Lithuania) was loaded in the first and last wells. The chamber was closed and run at 400 V and 100 mA for 30 min and DNA fragments were viewed by removing the gel slab from the tray and placed on a UV transilluminator, the Geldoc^™^ MP imaging system (Bio-Rad Laboratories, California, USA).

### 2.5 Detection of aflatoxin B1 in dairy cattle feeds

#### 2.5.1 Sample extraction

A 20 g particle size representative feed sample was prepared for extraction. An extraction solvent of 80% strength was prepared by adding 20 ml of distilled water to 80 ml of acetonitrile for each sample to be extracted. The prepared extraction solvent in the quantity of 100 ml was transferred to a container. The 20 g representative ground feed sample was then added, bringing the ratio of sample : solvent to 1:5 (w/v). The resulting mixture was blended and homogenized using Stomacher blender® for a minimum of 2 min. The mixture was allowed to settle and the extract filtered through Whatman No. 1 filter paper. The filtrate was collected in amber vials as extract for further analysis.

#### 2.5.2 AFB1 clean-up procedure using Immuno-affinity column (IAC)

A 5 ml aliquot of the extract was diluted with 14 ml of phosphate buffered saline (1 x PBS) solution (8.0 g NaCI, 1.2 g Na_2_HPO_4_, 0.2 g KH_2_PO_4_, 0.2 g KCI, dissolved in 990 ml purified water) and pH adjusted to 7.0 with HCI. The diluted filtrate (19 ml) which is equivalent to 1 g of sample was passed through the Aflatest^®^ IAC at a flow rate of 2 ml per minute to enable the aflatoxin captured by the antibodies present in the column. After that, the column was washed with 20 ml of 1 x PBS at a flow rate of 5 ml per minute in order to remove the unbound material, until air passed through the column. Aflatoxins were released from the column following elution with 1 ml of 100% methanol at a flow rate of 1 drop per second and 1 ml of water passed through the column and collected in the same vial to give a total of 2 ml. The eluate (AFs extract) was collected in an amber vials, evaporated to dryness with stream of nitrogen gas at 50 °C and stored at +4 °C.

#### 2.5.3 High performance liquid chromatography (HPLC)

Analysis on HPLC was performed to determine the exact concentrations of the extracted aflatoxins according to the method previously described [34] with modifications. The Shimadzu Prominence UFLC Liquid chromatography system (Kyoto, Japan) was used for the HPLC determination. It consists of a Liquid Chromatography, LC-20AD which is fitted to a degasser, DGU 20A_5R_, auto sampler (injection) SIL 20A, communication bus module CBM 20A, column oven CTO 20A, photodiode array detector SPD M20A and fluorescent detector RF 20A XL, connected to a gigabyte computer with Intel Core DUO and Microsoft XP operating system. The analytes that fluorescence was detected at specific excitation and emission wavelengths also referred to as the compound’s fluorescence signature. Extracts from IAC were dissolved in 500 μl of HPLC grade acetonitrile. Samples were run at a flow-rate of 1 ml per minute (min^−1^) retention times. Aflatoxin analysis involves the coupling to the detector a coring cell (CoBrA cell) (Dr Weber Consulting, Germany) as an electrochemical cell for the derivatisation of aflatoxins. The following mobile phases were used for the analysis of Aflatoxins- Methanol/Acetonitrile/Water (20/20/60, v/v/v) containing 119 mg of potassium bromide (KBr) and 350ul of nitric acid (4M HNO_3_).

#### 2.5.4 Recovery analysis

In order to confirm the effectiveness of the methods used for the extraction of aflatoxin B1, recovery analysis was carried out. Positive samples with known concentrations of aflatoxin B1 were spiked in triplicates with different concentrations of 5, 10 and 20 μgKg^−1^ of aflatoxin B1 standards (AFB1), mixed thoroughly and extracted. The resulting extracts were purified and analyzed with IAC and HPLC respectively in line with the methods described above. Percent recovery determined = (amount recovered divided by amount spiked) x 100.

## 3 Results

### 3.1 Occurrence of aflatoxigenic *Aspergillus flavus* in dairy feed samples

Out of the 48 isolates of *Aspergillus flavus* positively identified from 144 dairy cattle feeds collected across different diary settlements, 12 (approximately 25.0%) showed fluorescence reaction under a long ultra violet (UV) radiation, suggestive of aflatoxigenicity while the remaining 36 isolates of the same specie (making 75.0%) indicated negative reactions under UV, suggestive of non-toxigenic strains. Table 1 presents the occurrence and distribution of isolates of *Aspergillus flavus* across the different dairy settlements. The 12 aflatoxigenic strains showed 3 different fluorescence characteristics during their examination under a long UV (Table 2); indicating 3 aflatoxigenic strains as presented in Table 3. All the UV-positive isolates of the *Aspergillus flavus* possessed all the 4 tested aflatoxins encoding genes (*aflR, omt, ver* and *nor*) except one strain isolated from feeds of concentrates, which showed an amplicon band for *omt* only. This particular isolate showed the most strongest UV-fluorescence reaction, indicative of heavy aflatoxin production. However, the non-fluorescence reactors (representing the negative or the non-aflatoxigenic strains), possessed irregular amplicon banding patterns. Some displayed a complete 4 amplicon bands, indicative of potential aflatoxin production as presented in Table 3. DNA concentrations were determined as ng/μl and purified with the purity indices presented in Table 3. Partial sequencing of the intergenic spacer region, IGR enclosing *aflR-aflJ* was carried out on the 16 aflatoxigenic strains of *A. flavus* and *A. parasiticus* in order to identify specific nomenclature and the findings were presented in (Table 4).

**Table 1:** Occurrence and distribution of aflatoxigenic *A. flavus* and AFB1 in dairy cattle feeds among different dairy settlements.

Type of dairy settlement	No. of samples tested (N)	No. of feed samples tested positive for A. flavus, A (A/N%)	Occurrence of aflatoxigenic A. flavus, T (T/A%)	No. of feeds tested positive for AFB1, B (B/N%)	No. of AFB1 positive samples at critical concentrations, C (C/B%)			
					0.5-4.99 μgKg-1	5-10.99 μgKg-1	11-19.99 μgKg-1	≥ 20 μgKg-1
MIF	15	3	1	14	6	0	1	0
LIF	15	3	1	10	2	2	7	3
SIF	30	10	1	20	7	5	6	7
LCF	15	5	1	12	2	4	2	5
SCF	15	5	2	15	7	3	1	2
STFH	49	20	4	49	17	8	13	11
LTFH	2	0	0	2	2	0	0	0
MTFH	3	2	2	3	0	0	2	1
Total	144	48 (33.3)	12 (25.0)	125 (86.8)	42 (33.6)	22 (17.6)	32 (25.6)	29 (23.2)

MIF-Medium institutional farms (Medium, constitutes a cattle population of between 50–150 dairy cattle), LIF-Large institutional farm (Large, constitutes a cattle population of ≥ 150 dairy cattle), SIF-Small institutional farm (Small, constitutes a cattle population of < 50 dairy cattle), LCF-Large commercial farm, SCF-Small commercial farm, LTFH-represent large groups of traditional Fulani dairy herds, MTFH- represent medium groups of traditional Fulani dairy herds, STFH- represent small groups of traditional Fulani dairy herds.

**Table 2:** Occurrence of *A. flavus* and *A. parasiticus* isolates based on fluorescence characteristics.

Aspergillus spp	No. of feed samples tested, N	Total No. of isolates, n1 (n1/N)	No. of non-aflatoxigenic isolates, n2 (n2/N)	No. of aflatoxigenic isolates, n3 (n3/N)	Fluorescence characteristics of aflatoxigenic isolates			Total
					+	++	+++	
A. flavus	All samples	48	36	12	3	8	1	12
A. parasiticus	,,	16	12	4	0	4	0	4
Other Aspergillus spp	,,	22	22	0	0	0	0	0
Total	144	86 (59.7)	70 (81.4)	16 (18.6)	3	12	1	16

**Table 3:** Relationship between aflatoxin biosynthetic genes and UV characteristics of the aflatoxigenic strains of *Aspergillus flavus* and *A. parasiticus.*

Fluorescence characteristics	Isolate identity	Aflatoxins biosynthetic genes				Aflatoxin biosynthesis competency remarks	DNA concentration (ng/μl)	DNA purity index
		*aflR*	*omt*	*Ver*	*nor*			A260/A280
Very stong (+++)	No. 6	–	+	–	–	MC^+^/GC^+^	41.5	1.84
Strong (++)	,, 1	+	+	+	+	MC^+^/GC^+^	35.9	1.90
	,, 2	+	+	+	+	MC^+^/GC^+^	33.30	1.80
	,, 5	+	+	+	+	MC^+^/GC^+^	26.90	1.80
	,, 7	+	+	+	+	MC^+^/GC^+^	30.40	1.86
	,, 8	+	+	+	+	MC^+^/GC^+^	36.20	1.89
	,, 9	+	+	+	+	MC^+^/GC^+^	31.50	1.84
	,, 10	+	+	+	+	MC^+^/GC^+^	28.10	1.80
	,, 11	+	+	+	+	MC^+^/GC^+^	45.60	1.82
	,, 12	+	+	+	+	MC^+^/GC^+^	46.10	1.76
	,, 13	+	+	+	+	MC^+^/GC^+^	78.10	1.79
	,, 15	+	+	+	+	MC^+^/GC^+^	115.60	1.82
	,, 16	+	+	+	+	MC^+^/GC^+^	49.00	1.76
Weak (+)	,, 3	+	+	+	+	MC^+^/GC^+^	34.40	1.80
	,, 4	+	+	+	+	MC^+^/GC^+^	12.90	1.85
	,, 14	+	+	+	+	MC^+^/GC^+^	30.80	1.87
Negative (-)	Nos. 17-37	+	+	+	+	MC^−^/GC^+^	74.80-89.00	1.76-1.86
	,, 38-46	+	+	–	+	MC^−^/GC^−^	48.5-64.64	1.82-1.89
	,, 47-55	+	+	+	–	MC^−^/GC^−^	42.90-52.54	1.79-1.87
	,, 56-64	+	–	+	+	MC^−^/GC^−^	40.40-48.90	1.83-1.86

MC^+^/GC^+^ = Microbiologically and genetically competent, MC^−^/GC^+^ = Microbiologically incompetent but genetically competent, MC^−^/GC- = Microbiologically and genetically incompetent.

**Table 4:** Sequenced aflatoxigenic strains of *A. flavus.*

	Isolate No.	Specific genes for differentiation between *A. flavus* and *A. parasiticus*			Specific nomenclature	Strain identification based on partial sequencing	
		*IGS(aflR-aflJ)*	*IGS/BgIII restriction sites*	*ITS(fla)*		Identified strain	Accession No.
1	No. 6	+	2	+	*flavus*	EGY 1	KM870530.1
2	,, 1	+	2	+	*flavus*	ITD-G11	KM057751.1
3	,, 2	+	2	+	*flavus*	EGY 1	KM870530.1
4	,, 5	+	2	+	*flavus*	MJ49	HM590660.1
5	,, 7	+	1	–	*A. parasiticus*	–	–
6	,, 8	+	1	–	*A.parasiticus*	–	–
7	,, 9	+	2	+	*flavus*	EGY 1	KM870530.1
8	,, 10	+	2	+	*flavus*	HKF49	HM773231.1
9	,, 11	+	2	+	*flavus*	TZ 1985	GU953210.1
10	,, 12	+	2	+	*flavus*	HKF 13	HM773227.1
11	,, 13	+	1	–	*A. parasiticus*	–	–
12	,, 15	+	1	–	*A. parasiticus*	–	–
13	,, 16	+	2	+	*flavus*	ITD-G11	KM057751.1
14	,, 3	+	2	+	*flavus*	HKF 30	HM773230.1
15	,, 4	+	2	+	*flavus*	EGY 1	KM870530.1
16	,, 14	+	2	+	*flavus*	EGY 1	KM870530.1
Control organisms:							
17	*A. flavus*	+	2	+	Ctrl + ve 1	–	
18	*A. parasiticus*	+	1	–	Ctrl + ve 2	–	

### 3.2 Comparative occurrence and distribution of aflatoxigenic and non-toxigenic *A. Flavus*

The study revealed that the occurrence of non-aflatoxigenic strains was significantly higher (P < 0.05) than the aflatoxigenic strains in the various feed types (Table 5). Both fresh and preserved feeds analyzed in the study showed lower incidences of aflatoxigenic strains of the *A. flavus* (Table 6). Findings in this study revealed a significant difference in the occurrence of the aflatoxigenic strains of *A. flavus* among the stored dairy cattle feeds (P < 0.05) when compared with the feeds obtained as fresh samples (Table 6). Statistically significant difference (p < 0.05) was also noticed between the concentrate fortified feeds, feeds of grain origin and that of dry pasture (Table 6). Proportion of occurrence of aflatoxigenic *A. flavus* and the associated moisture contents amongst the various feed types also showed statistically significant difference (p < 0.05) between the concentrate fortified feeds and the dry pasture (Table 5).

**Table 5:** Relationship between Feed moisture contents and occurrence of *A. flavus* and AFB1 in various feed types.

Feed type	No. of feed samples tested, N	Mean moisture contents (%)	Occurrence of A. flavus, A(A/N%)	No. of Non-aflatoxigenic isolates, B(B/A%)	No. of aflatoxigenic isolates, C (C/A%)	No. of AFB1 positive samples, n (n/N%)
Feed + concentrates	69	6.0 ± 1.3a	23 (52.1)	16	7	69
Feeds of grain origin	60	4.3 ± 1.0a	21 (39.6)	17	4	47
Dry pasture	15	2.5 ± 0.9c	4 (8.3)	3	1	9
Total	144	–	48 (33.3)	36 (75.0)	12 (25.0)	125 (86.8)

**Table 6:** Distribution of aflatoxigenic *A. flavus* and AFB1 contaminated feeds in relation to feed types, feed category.

Feed types and condition of storage	No. of sample tested for AFB1 (N)	No. tested positive for aflatoxigenic A. flavus, n1 (n1/N%)	No. of AFB1 positive samples, n2 (n2/N%)		AFB1 concentration range (μgKg-1)	AFB1 total mean concentration (μgKg-1)	No. of AFB1 positive samples, n3 (n3/n2%) at critical concentrations				
							0.5-4.99 μgKg-1	5–10.99 μgKg-1	11-19.99 μgKg-1		≥ 20 μgKg-1
		Types of feeds									
Feeds + concentrates	69	7	60		2.6–24.8	11.5 ± 8.0	16	10		14	19
Feeds of grain origin only	60	4	56		2.6-23.6	9.8 ± 7.0	18	11		17	10
Dry pasture	15	1	9		0.5–12.1	1.8 ± 3.0	8	1		1	0
Total	144	12 (8.3)	125 (86.8%)		0.5 – 24.8	7.7 ± 0.6	42 (33.6%)	22 (17.6%)		32 (25.6%)	29 (23.2%)
		Feed category									
Fresh feeds	81	8	72	0.5 – 23.8		7.6 ± 7.0	28	19		17	8
Stored feeds	63	4	53	3.3 – 24.8		13.5 ± 8.0	16	3		14	20
Total	144	12 (8.3)	125 (86.8)	0.5 – 24.8		10.5 ± 8.0	44(35.2%)	22(17.6%)		31 (24.8%)	28(22.4%)

### 3.3 Occurrence of aflatoxins B1 in diary feeds

**Fig. 1:**
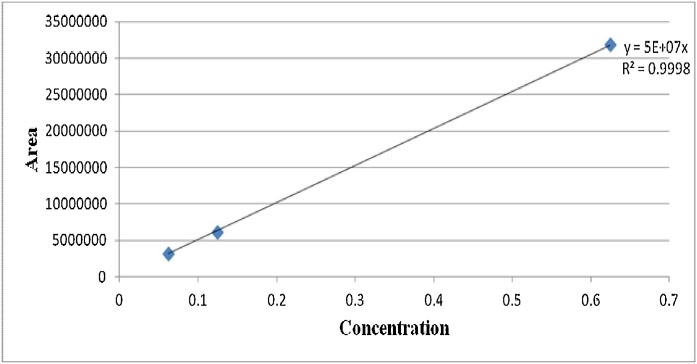
Standard curve for AFB1.

**Fig. 2:**
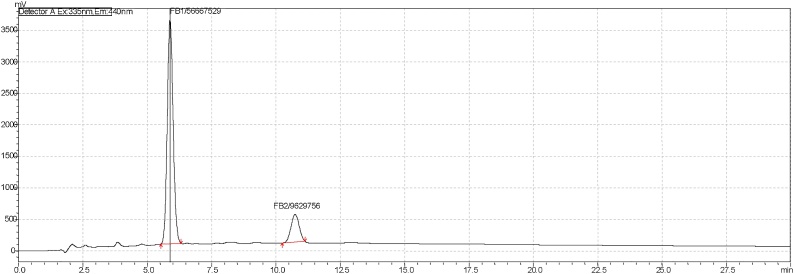
Illustration of a chromatogram of dairy cattle feed sample from the largest institutional farm at 20 ul injection on high performance liquid chromatography coupled with RF detector and KobrA cell.

**Fig. 3:**
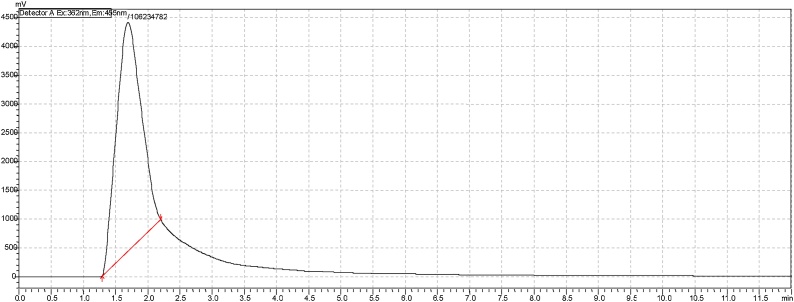
Illustration of a chromatogram of AFB1 non-contaminated dairy cattle feed sample at 20 ul injection on high performance liquid chromatography coupled with RF detector and KobrA cell.

**Fig. 4:**
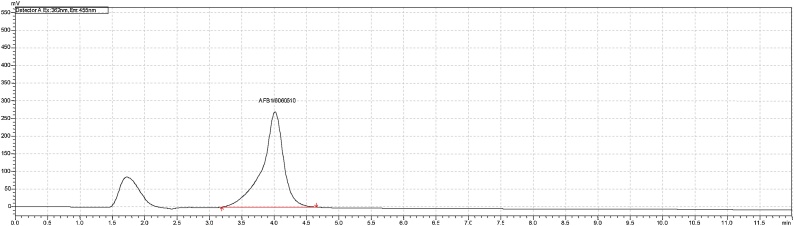
Illustration of a chromatogram of dairy feed sample from traditional Fulani dairy herd groups at 20 ul injection on high performance liquid chromatography coupled with RF detector and KobrA cell.

Positive aflatoxin B1 (AFB1) samples were detected and concentrations determined by extrapolation using the equation, y = 5E + 07x as shown in (Fig. 1). Spiked AFB1 samples showed recovery values in the range of 70–110% (mean recovery rate of 89.6%), with limits of detection (LOD) and quantification (LOQ) being 0.18 μg Kg-1 and 0.53 μg Kg-1 respectively. Out of 144 dairy cattle feed samples collected for testing using HPLC analytical method, a total of 125 (86.8%) samples tested positive for aflatoxins B1 (Table 7). Of these positive samples, 83 (66.4%), showed levels of AFB1 contaminations exceeding the minimal acceptable levels; of which 22 (26.5%), 32 (38.6%) and 29 (34.9%) had AFB1 contamination levels of between 5 and 24.8 μgKg-1 (Table 7). Fig. 2, Fig. 3, Fig. 4 are illustrations of chromatograms resulting from the analysis of dairy cattle feeds for AFB1 using HPLC coupled with KobraA cell for derivatization and RF detectors for detecting fluorescent compounds.

**Table 7:** Distribution of aflatoxigenic *A. flavus* and AFB1 positive samples in relation to farm type.

	No. of samples tested, N	No. tested positive for aflatoxigenic A. flavus, n1 (n1/N%)	No. tested positive for AFB1, n2 (n2/N%)	AFB1 concentration range (μgKg-1)	AFB1 total mean concentration (μgKg-1)	No. of AFB1 positive samples, n3 (n3/n2%) at critical concentrations			
						0.5-4.99 μgKg-1	5-10.99 μgKg-1	11-19.99 μgKg-1	≥ 20 μgKg-1
Institutional farm	60	3	44	0.78 – 24.5	7.7 ± 8.1	15	7	14	10
Commercial farm	30	3	27	2.6 – 23.8	12.0 ± 7.6	9	7	3	7
Traditional Fulani dairy herds	54	6	54	0.5 – 24.8	10.8 ± 7.6	18	8	15	12
Total	144	12 (8.3)	125 (86.8)	0.5 – 24.8	13.6 ± 7.8	42 (33.6)	22 (17.6)	32 (25.6)	29 (23.2)

### 3.4 Occurrence and distribution of aflatoxigenic *A. flavus and aflatoxin B1*

An indirect relationship was observed between the occurrence of aflatoxigenic *A. flavus* and its metabolite, AFB1. Lower incidences of the aflatoxigenic strains gave rise to unprecedented higher AFB1 positive samples as shown in (Table 7). Feed samples collected mostly from traditional Fulani dairy herds and other small scale dairy commercial farms showed significant positive indices for the contaminations due to aflatoxigenic *A. flavus* strains and their metabolite, AFB1 (Table 8). The number of AFB1 positive feed samples at critical concentrations exceeding the maximum acceptable limits of (5–10 and 10–20) μgKg-1 in both dairy and other productions respectively, was significantly higher amongst the dairy farms belonging to traditional Fulani and small scale commercial farms (Table 8). However, small scale dairy farms belonging to institutional settlement where the management system involved modified free range characterized by dry pasture and skeletal supplementation, were found to possess extremely low positive indices for both cases of aflatoxigenic *A. flavus* and AFB1 (Table 8).

**Table 8:** Relationship in the occurrence and distribution of the aflatoxigenic strains of *A. flavus* and AFB1.

Dairy settlements	Management system	No. of feed samples tested (N)	No. positive for aflatoxigenic A. flavus (n1)	Positive index fraction (n1/N)	No. positive for AFB1 (n2)	Positive index fraction (n2/N)	No. of AFB1 samples at critical concentrations (μgKg-1)			
							0.5-4.99	5-10.99	11-19.99	≥20
LIF	SIFS	15	1	0.07	10	0.67	10	0	0	0
MIF	SIFS	15	1	0.07	14	0.93	0	3	6	5
SIF	MFRSDP	15	0	0.00	7	0.50	4	1	2	0
LCF	SIFS	15	1	0.07	12	0.80	1	4	5	2
SCF	SIFS	15	2	0.13	15	1.00	3	2	4	6
LTFH	FRSS	2	0	0.00	2	1.00	0	0	1	1
MTFH	FRSS	3	2	0.67	3	1.00	0	0	1	2
SIF	SIFS	15	1	0.07	13	0.87	4	3	2	4
STFH	FRSS	49	4	0.08	49	1.00	20	9	11	9
Total	–	144	12	–	125	–	42	22	32	29

MIF-Medium institutional farms, LIF-Large institutional farm, SIF-Small institutional farm, LCF-Large commercial farm, SCF-Small commercial farm, LTFH - Large traditional Fulani dairy herds; MTFH – Medium traditional Fulani dairy herds; STFH – Small traditional Fulani dairy herds; SIFS-Semi-intensive + feed supplementation, MFRSDP-Modified free range subsisting only on dry pasture, FRSS- Free range + skeletal supplementation.

### 3.5 The effect of farm size on the occurrence and distribution of AFB1

The effect of farm size on the level of cataminations due to aflatoxigenic A. flavus and its toxin, AFB1 is presented in Table 9. Small scale dairy farms of cattle population less than 50 showed the highest feed samples of 84(67.2%) with detectable levels of AFB1 out of which 53 (63.1%) samples showed a detectable AFB1 concentration levels of between 5 and ≥20 μgKg-1 (Table 9). However, large and medium dairy farms having dairy cattle populations of greater than 150 and between 50–150 respectively showed lower proportions of detectable AFB1 contaminated samples, with relatively significant proportions of samples with AFB1 exceeding the acceptable levels of AFB1 as presented in Table 9.

**Table 9:** Occurrence and distribution of aflatoxigenic *A. flavus* and AFB1 in relation to farm size.

Size of dairy farms and herds	No. of feed samples tested, N	No. tested positive for aflatoxigenic A. flavus, n1 (n1/N%)	No. of AFB1 positive samples, n2 (n2/N%)	AFB1 concentration range (μgKg-1)	AFB1 total mean concentration (μgKg-1)	No. of AFB1 positive samples, n3 (n3/n2%) at critical concentrations			
						0.5 – 4 μgKg-1	5– 10.99 μgKg-1	11-19.99 μgKg-1	≥ 20 μgKg-1
LCP (> 150)	32	3	24	3.1-23.7	7.4 ± 7.0	11	4	6	3
MCP (50 – 150)	18	3	17	5.4-24.8	16.1 ± 7.0	0	3	7	7
SCP (< 50)	94	6	84	0.5-24.5	9.4 ± 8.0	31	15	19	19
Total	144	12 (8.3%)	125 (86.8%)	0.5-24.8	11.0 ± 7.3	42 (33.6)	22 (17.6)	32 (25.6)	29 (23.2)

LCP-Large cattle population, MCP-Medium cattle population, SCP-Small cattle population.

### 3.6 The effect of feed type on the occurrence and distribution of AFB1

Table 6, presents the commonly used dairy cattle feed samples in relation to their ability to support aflatoxin production. The result showed a close range in the mean AFB1 concentrations particularly between feeds fortified with concentrates and feeds of grain origin (P > 0.05). However, feeds of dry pasture particularly dry grasses and stalks without cobs, showed a significantly lower AFB1 mean concentration (P < 0.05) when compared with the other feed types. A significant (P < 0.05) proportions of feeds fortified with concentrates and feeds of grain origin were observed to show AFB1 detectable level exceeding the recommended allowable limits of less than 5 μgKg-1 in dairy production when compared with feeds of dry pasture that had very few samples exceeding the set limits.

### 3.7 The effect of farm type on the occurrence and distribution of AFB1

Table 7 displays the contamination levels of AFB1 according to the different types of farm (i.e. institutional, commercial or Fulani herd groups). There was no statistically significant difference (p > 0.05) in AFB1 mean concentrations between the different types of dairy farms. However, AFB1 mean concentration in Fulani dairy herd groups was apparently seen to be lower than either of the commercial or institutional farm which had comparatively higher levels of AFB1 concentrations. Importantly, in the study, all the 54 feed samples from the traditional Fulani Dairy herd groups showed detectable levels of AFB1, of which 32 feed samples showed detectable levels of AFB1 exceeding the critical concentrations of 5–10 μgKg-1 and 10- 20μgKg-1 for dairy and other productions respectively.

### 3.8 Effect of feed category (either freshly formulated and used or purchased and preserved) on the occurrence and distribution of AFB1

Table 6, also presents a distribution pattern of detectable levels of AFB1 between the two category of feed samples (fresh and preserved) used in this study. On the average, there was statistically significant difference (P < 0.05) between the two groups in relation to the mean AFB1 concentrations and the number of aflatoxigenic *A. flavus* strains. Both fresh and preserved feeds showed significant proportions of feed samples with detectable levels of AFB1 exceeding the critical concentrations of 5–20 for both dairy and other animal productions.

## 4 Discussion

This research was set out to provide answer to the erroneous perception that all *Aspergillus flavus* contaminants were toxigenically important. This is very key considering the Nigerian situation where dairy production occupies a monumental niche in the agricultural sector. Dairy cattle feeds, in this respect, may represent suitable media for the growth and proliferation of *A. flavus* and its toxin (AFB1). The major risk amongst others, lies in the metabolic conversion of AFB1, a known hepato-cellular carcinogen, to a deleterious metabolite, AFM1, which is excreted into the milk following the consumption of contaminated feed by the cattle [17]. In the current study, high level of occurrence of *Aspergillus flavus* in most feed types was observed indicating the suitability of the diverse feed compositions and the environmental conditions for their growth and proliferation. [33] demonstrated the role of moisture in fungal growth. He reported an optimal moisture level of 14% and above for the growth and proliferation of fungi. The current study, in a way, reveals findings suggestive of fast ecological adaptation particularly with regard to *A. flavus* group. Findings from this study showed that moisture content as low as 2.28% yielded significant growth of the fungal group. It thus implies that the said organism of high economic pedigree, possesses strong ecological competence in competing with other organisms within its niche. This may fashion a better reason for its high incidences reported in some other works [23,11]. However, in the current study also a relatively low occurrence of the aflatoxigenic strains of *Aspergillus flavus* (13.9% of the 86 isolates of Aspergilli, out of which 48 are *A. flavus*) was observed. This relatively low incidence of the toxigenic strains may be perceived as though non-significant, the instability and the genetic diversity shrouding the biosynthesis of aflatoxins, may raise safety concerns. This is very important considering the very high incidence of AFB1 (86.8%) seen in association with the low occurrence of the toxigenic strains of the *A. flavus* found in the study. This finding is in agreement with a previous report of [20] who articulated a correlation between lower incidence of aflatoxigenic strains of *Aspergillus flavus* and the unpredictable high levels of AFB1 produced by the agent. Of interest in this study is the variable strength of the aflatoxin produced by the aflatoxigenic strains when viewed under a long wavelength of ultra violet light. Dairy cattle feeds fortified with concentrates and grains showed higher incidences of aflatoxigenic *A. flavus* strains and higher concentrations of aflatoxin B1. Previous findings only associated higher incidences of aflatoxin production with certain enriched feedstuff [7,57] but the current finding in addition also showed the influence of enriched concentrates on the concentration of the aflatoxins produced. This finding is very important as it may suggest the importance of nutrition in gene expression. This may explain the ‘very strong’ and ‘strong’ UV characteristic features seen among the aflatoxigenic isolates cultured from feeds of concentrate origin. It is consequently believed that poor nutrition may temporarily suppress certain strains of *A. flavus* from expressing their full toxigenic strength even though they possess genetic competence. This may further explain reason why most of the UV-negative samples found in this study, still possess the toxigenic competence due to the presence of a quadruplet band indicating the availability of the 4 biosynthetic genes which encode for functional products. This finding strengthens and puts in better perspective the previous explanations of [4,44] that some aflatoxin-producing strains may fail to express aflatoxin in their final phase of production. It is also believed that poor nutrition, as an environmental factor, may cause genetic instability resulting in simple mutations (substitution of some bases) leading to the formation of non-functional products [13,47]. This aspect of our findings portends public health risk with respect to the advocated use of the “non-toxigenic” strains of *Aspergillus flavus* for biological control of mycotoxin contamination of crops [26,18].

Partial sequencing of the amplified *IGS* regions successfully identified some diverse aflatoxigenic strains of *A. flavus associated* with the sampled dairy cattle feeds in Nigeria. The strains identified were *A. flavus* EGY1, *A. flavus* ITD-G11, *A. flavus* MJ49, A*. flavus* HKF30, *A. flavus* HKF13, *A. flavus* HKF49 and *A. flavus* 1985. The finding is an indication of strain diversity in the web of fungal contamination of feeds. This agrees with the previous work [26,18]. This diversity may also reflect the risks associated with the unregulated international trades especially within Africa where many traders of animal feed ingredients engage in trading through illegal routes. However, the utilized intergenic spacer region for strain identification in this study lacked technical competence in differentiating between the strains of *A. flavus* and *A. parasiticus.* The close genomic similarity that exists between the two species may be attributed to this. Therefore, an extended genomic sequencing may be required to clear this complexity. Other gene biomarkers used to target the flanking gene located on the highly variable and specific intergenic region of the fungus and the *aflR-aflJ* intergenic region fragments using restriction endonuclease, *Bg III* achieved the objective of differentiating between the two closely related species [8].

Mycototoxins which are produced largely by fungi have accounted for high economic losses through reduced animal production, trade barriers for consumable food items and direct loss of lives [10] as reported by [20]. Increasing incidence of aflatoxins has dominated the health concerns of people due to its high carcinogenic potential; documented across the globe [39,14,53]; Africa [19] and Nigeria [45,49]. In particular AFB1, a known second largest cause of liver cancer, when ingested by ruminants, about 1–2% of it is converted to its metabolite, aflatoxin M1 which is majorly excreted in milk [23]. AFM1 has been incriminated to have as high carcinogenic potency as the parent compound, AFB1 [36]. In effect, it implies that the synergistic effect of both AFB1 and AFM1 may bear worst economic implications on animal health and production on one hand and human health on the other hand. Few amongst other health implications reported include carcinogenic, teratogenic, hepatogenic, mutagenic and immunosuppressive effects in animals and man. These clinical effects coupled with anemia-a prominent clinical feature of aflatoxicosis in dairy cattle, have led to severe milk drop due to reduced feed intake by the affected animals [29].

Dairy cattle feeds collected and analyzed in this study showed a low (8.3%) and high (86.8%) incidences of aflatoxigenic *A. flavus* and detectable levels of AFB1 respectively. This finding is partly in agreement with the report of [20] which demonstrated an AFB1 incidence as high as 91.7% across dairy samples tested for aflatoxins. The observed disproportionate incidences between the aflatoxigenic *A. flavus* and its metabolite, AFB1, may be due to rapid depletion of the vegetative phase of the organism due to harsh environmental conditions. The harsh environmental conditions may not show any significant effect on its metabolite, AFB1 due to its thermo-stability. Significant proportions of these feed samples (66.4%) showed considerable detectable levels of AFB1 within the critical concentration ranges of 5–10 and 10–20 μgKg-1. These findings agree with the report of [57] in another continent and have demonstrated considerable levels of contravention exceeding the AFB1 acceptable limits of 5 μgKg-1 and 10 μgKg-1 set by the EU and USFDA respectively for dairy industries. Higher proportions of these feed samples with AFB1 concentrations exceeding the limit of contravention, were associated with preserved feeds of grain origin and feeds fortified with concentrates. Majority of these samples were from traditional Fulani dairy herds and some other categories of dairy farms operating semi-intensive management system. These findings may be suggestive of the critical impacts of feed supplementation on the found incidences of the aflatoxigenic *A. flavus* and AFB1 respectively [17]. In another finding in the same study, dairy cattle placed on the same management system but differ in their feeding regimen, such as dry pasture, showed low positive index fractions for both *A. flavus* and AFB1. This further strengthened the claim that feeds of higher nutritional status may influence the level of AFB1 contamination in feeds. Feeds of dry pasture, particularly the leaves and stalks, showed low AFB1 level when compared with the cobs and the grains [9,57]. This may explain the relatively low level of AFB1 concentrations in the grass samples analyzed in this study.

The significant difference seen in the mean concentrations of AFB1 between small scale dairy farm encompassing the traditional Fulani dairy cattle herds and other farms of conventional dairy farms may be attributed to poor husbandry management system in terms of processing and storage of feeds [28]. This may explain the high positive index fraction observed amongst the traditional Fulani herds and other small scale dairy farms. In this study, preserved feeds showed a statistically significant AFB1 mean contamination level (P > 0.05) than the mean AFB1 contamination levels of the fresh feeds. This is a clear demonstration of the effect of poor storage of feeds below the optimal level of feed storage on the growth and proliferation of *A. flavus* group [3].

## 5 Conclusion

Findings from this study showed a high level of risk associated with the consumption of contaminated feeds by dairy cattle during the active phase of milk production. This was supported by the fact that aflatoxigenic *Aspergillus flavus* and its major metabolite, AFB1, were significantly present as contaminants in feeds fed to dairy cattle in the active phase of milk production in Kaduna State, Nigeria. The associated risks became more worrisome when it was found that a multiplying incidences of AFB1 were observed to be associated with low incident rates of aflatoxigenic strains of *A. flavus.* These contaminants were detected from dairy cattle feeds collected from both traditional Fulani dairy herds and the conventional dairy farms. Higher risks were found to be associated with the traditional dairy settings than the conventional ones. Conventional dairy farms may also possess health risks if feed supplements were not properly preserved. Higher incidences of heavy AFB1 contamination were observed among feeds of concentrate and grain origins than the usual dry pasture. AFB1 has been known to be the second most important hepato-cellular carcinogen in humans. This agent metabolizes in the body of animal and releases its metabolite, AFM1, which is as important as the parent compound, into the milk in the udder. Findings in this study also showed that the critical allowable limits set by the international regulatory bodies, EU and USFDA, were in many instances, contravened by most dairy outfits, thereby exposing the consumers of dairy products to the risks of acute or chronic aflatoxicosis and colossal production losses through drops in dairy output and international trade. Findings from this study also showed that reasonable proportion of *A. flavus* contaminants was not toxigenic and the few identified aflatoxigenic strains displayed enormous aflatoxin-producing capabilities. Government should empower the relevant professional bodies for better regulatory procedures through active and all-inclusive legislations and policies.

## Conflict of interest

Authors of this manuscript declare no conflict of interest.

## Transparency document

Transparency document
